# Digital workflow with intraoral scanning and CAD/CAM PMMA crowns in a non-cooperative pediatric patient with early childhood caries: a case report

**DOI:** 10.3389/froh.2026.1848861

**Published:** 2026-05-22

**Authors:** Claudia Reytor-González, Viviana Guachamin-Catota, Anahí Hilen Carrillo Zúñiga, Marco Andrés González Hidalgo, Náthaly Mercedes Román-Galeano, Daniel Simancas-Racines

**Affiliations:** 1 Facultad de Ciencias de la Salud y Bienestar Humano, Universidad Tecnológica Indoamérica, Ambato, Ecuador; 2 Facultad de Odontología, Universidad San Francisco de Quito, Quito, Ecuador; 3 Facultad de Ciencias Médicas, de la Salud y la Vida, Universidad Internacional del Ecuador UIDE, Quito, Ecuador

**Keywords:** CAD/CAM crowns, child oral health, digital workflow, early childhood caries, intraoral scanning, pediatric dentistry

## Abstract

Early childhood caries can cause extensive coronal destruction in preschool children, often requiring complex rehabilitation at an age when cooperation is limited. This case report describes a behavior-adapted digital workflow used to complete full-mouth rehabilitation in a four-year-old boy with severe early childhood caries and very limited cooperation. Clinical and radiographic assessment showed advanced multi-tooth carious involvement, with marked destruction of the maxillary anterior segment. Differential diagnostic reasoning supported severe early childhood caries as the most consistent diagnosis. Because conventional impression-taking was not tolerated, a two-step intraoral scanning workflow was used to support treatment planning and definitive anterior rehabilitation. Management included disease control, caregiver education, sealants, restorative procedures, pulpectomies where indicated, post-and-core reconstruction, and full-coverage restorations. In the maxillary anterior segment, biologic posts were used for severely destroyed primary incisors, followed by computer-aided design and computer-aided manufacturing fabrication of polymethyl methacrylate crowns. The crowns were tried in, adjusted, and cemented under controlled isolation. No pain or immediate complications were observed. At the six-month follow-up, the restorations remained intact, gingival tissues were healthy, occlusion was clinically acceptable, and periapical findings were unremarkable, with no radiographic signs suggestive of pathological resorption. This case suggests that intraoral scanning and digital restorative workflows may provide a practical pathway for completing definitive rehabilitation in selected non-cooperative pediatric patients, provided that disease control, isolation, fit verification, cementation quality, and structured follow-up are maintained.

## Introduction

Early childhood caries (ECC) remains a major challenge in pediatric dentistry because it can begin soon after tooth eruption, progress rapidly in high-risk children, and often remain untreated, with consequences that extend beyond pain and infection to measurable impacts on the child's and family's quality of life (1, 2). In preschool-aged children, anterior tooth destruction can be particularly consequential because it affects function, appearance, and family wellbeing, and systematic evidence consistently links ECC severity with poorer oral health–related quality of life in both children and caregivers (1, 3).

Rehabilitation frequently requires multiple steps and high procedural tolerance. Conventional impressions may trigger gag reflex, anxiety, or refusal—especially in non-cooperative children—becoming a practical barrier to completing indirect restorations (4). Intraoral scanning and digital workflows have increasingly been evaluated in pediatric settings, with systematic evidence indicating that children often report higher comfort and preference for digital impressions compared with conventional techniques (5). Although digital workflows are widely adopted in adult prosthodontics, their implementation in young children is less frequent, in part because of cost and resource constraints; clinically, however, these tools may be particularly valuable when conventional impressions are not feasible.

This report presents a behavior-adapted, scanner-based workflow used to complete full-mouth rehabilitation in a four-year-old child with severe ECC and very limited cooperation (Frankl 1), in whom conventional impression-taking was not tolerated. Although intraoral scanning has been increasingly reported in pediatric dentistry, the additional contribution of this case lies in documenting its use as a practical strategy to overcome a behavioral barrier that threatened treatment completion. By detailing a two-step intraoral scanning approach, CAD/CAM fabrication of definitive anterior crowns, and structured follow-up, this case provides practice-oriented insight into how digital workflows may support definitive restorative care in highly uncooperative pediatric patients.

## Patient information

A four-year-old male with limited cooperation (Frankl 1) was brought by his mother, who expressed concern about his current oral health. Medical history was unremarkable. Dietary history was notable for frequent sugar exposure. According to the caregiver report at presentation, the child was brushing independently, and oral hygiene was inadequate. Atypical swallowing was also reported.

## Clinical findings

Clinical examination revealed a severe, generalized caries burden with extensive coronal destruction, most pronounced in the primary maxillary anterior region. Multiple teeth presented large cavitated lesions with substantial loss of tooth structure, compromising anterior function and esthetics. The distribution and severity of the lesions were consistent with advanced ECC and indicated the need for comprehensive preventive and restorative management.

## Diagnostic assessment

**Figure 1:**
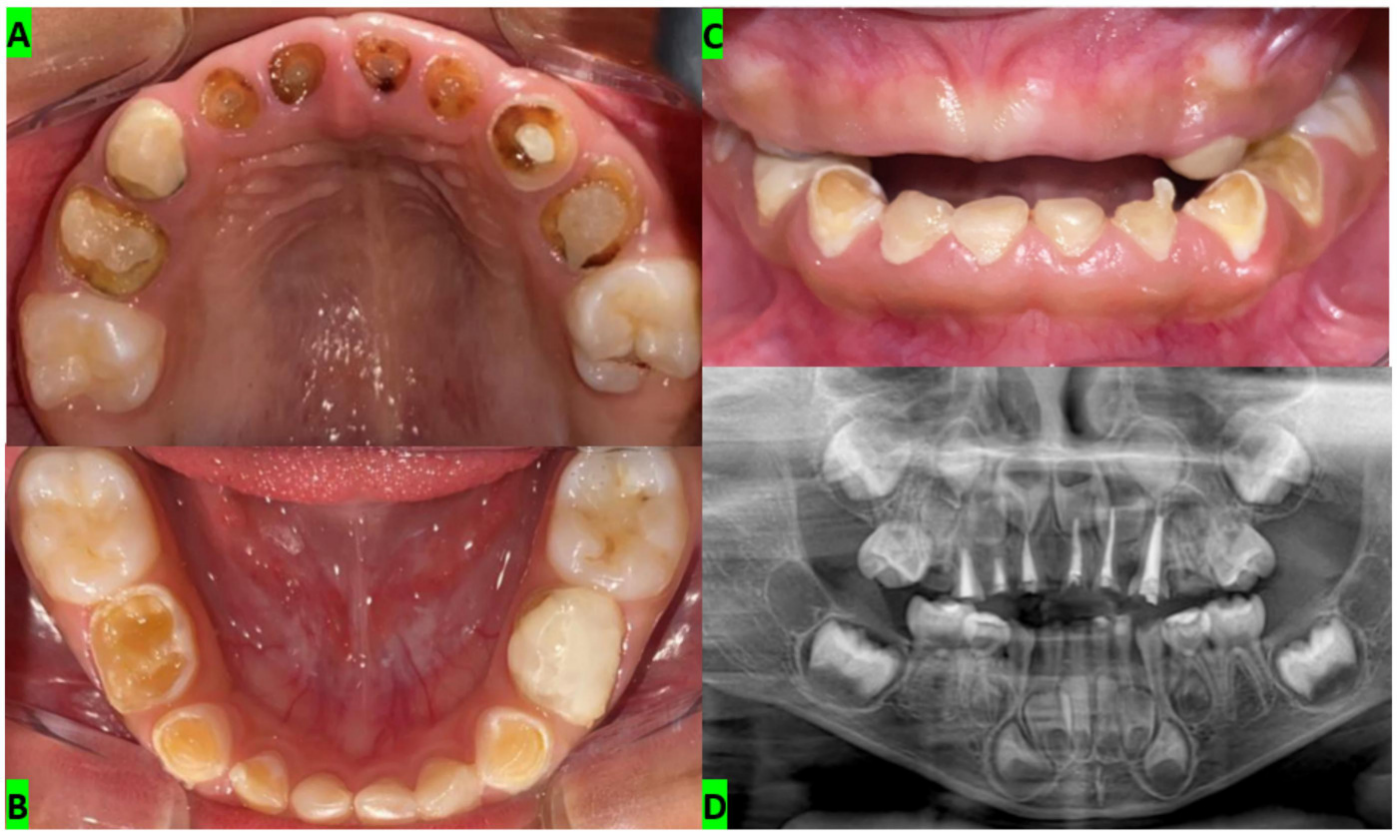
Baseline clinical and radiographic findings consistent with early childhood caries. **(A)** Maxillary occlusal view showing extensive cavitated carious lesions and marked coronal breakdown affecting multiple primary teeth, particularly the anterior segment. **(B)** Mandibular occlusal view demonstrating additional carious involvement and loss of tooth structure in the primary dentition. **(C)** Frontal intraoral view illustrating anterior tooth destruction and compromised function/esthetics at presentation. **(D)** Panoramic radiograph obtained at baseline to assess overall disease extent and support comprehensive treatment planning.

The diagnosis of ECC was established based on clinical and radiographic evaluation (Figure 1). Clinical caries staging was recorded using the International Caries Detection and Assessment System (ICDAS), and radiographic lesion depth was staged using the International Caries Classification and Management System (ICCMS) radiographic scoring system (RA–RC) according to the extent of radiolucency. A panoramic radiograph was obtained to assess disease extent and support treatment planning. The patient's behavior was classified as Frankl 1, representing a major practical constraint for diagnostic and restorative procedures.

Tooth-level assessment demonstrated advanced anterior maxillary involvement: teeth 51, 52, 53, 61, and 62 presented extensive cavitated lesions (ICDAS 6) with deep radiographic involvement staged as RC5, and tooth 63 showed advanced involvement (ICDAS 5; RC5). Radiographs also demonstrated pre-existing pulpectomy treatments in teeth 51, 52, 53, 61, 62, and 63 (Table 1). Despite prior endodontic therapy, severe coronal breakdown and findings consistent with loss of coronal seal suggested microleakage and contamination, informing the need for comprehensive re-intervention and definitive full-coverage rehabilitation.

**Table 1:** Tooth-level details and treatment performed.

Tooth	ICDAS	ICDAS/ICCMS radiographic scoring system	Endodontic treatment	Treatment performed
51	6	RC5	Pulpectomy	Biologic post+core build-up (Paracore) + CAD/CAM PMMA crown
52	6	RC5	Pulpectomy	Biologic post+core build-up (Paracore) + CAD/CAM PMMA crown
53	6	RC5	Pulpectomy	Celluloid crown
54	6	RB4	None	Celluloid crown
55	2	RA1	None	Sealant
61	6	RC5	Pulpectomy	Biologic post+core build-up (Paracore) + CAD/CAM PMMA crown
62	6	RC5	Pulpectomy	Biologic post+core build-up (Paracore) + CAD/CAM PMMA crown
63	5	RC5	Pulpectomy	Fiber post+celluloid crown
64	5	RB4	None	Celluloid crown
65	1	RA1	None	Sealant
71	1	RA2	None	Celluloid crown
72	1	RA2	None	Celluloid crown
73	4	RA2	None	Celluloid crown
74	4	RA3	None	Celluloid crown
75	2	RA1	None	Sealant
81	1	RA2	None	Celluloid crown
82	3	RA2	None	Celluloid crown
83	4	RA3	None	Celluloid crown
84	5	RB4	None	Celluloid crown
85	1	RA1	None	Sealant

ICDAS, International Caries Detection and Assessment System (clinical caries scoring). ICCMS, International Caries Classification and Management System; radiographic depth was staged using ICCMS radiographic scores (RA–RC) based on the extent of radiolucency. PMMA, polymethyl methacrylate. CAD/CAM, computer-aided design/computer-aided manufacturing.

Differential diagnostic reasoning considered other potential causes of extensive tooth structure loss in the primary dentition, including developmental enamel defects, traumatic dental injury, and non-carious tooth wear. These conditions were considered less likely because the lesions showed a cavitated carious pattern, affected multiple teeth, and were consistent with the caregiver-reported history of frequent sugar exposure and inadequate oral hygiene. Overall, the clinical distribution, lesion morphology, radiographic findings, and risk profile supported the diagnosis of severe ECC.

## Timeline

The clinical sequence was organized as a staged rehabilitation pathway adapted to the child's limited cooperation and extensive caries burden. Initial assessment included caregiver interview, dietary and oral-hygiene evaluation, clinical examination, radiographic assessment, and tooth-level caries staging using ICDAS and ICCMS. Based on the severity of early childhood caries and the degree of coronal destruction, a full-mouth rehabilitation plan was established, including preventive, restorative, endodontic, and full-coverage procedures.

**Figure 2:**
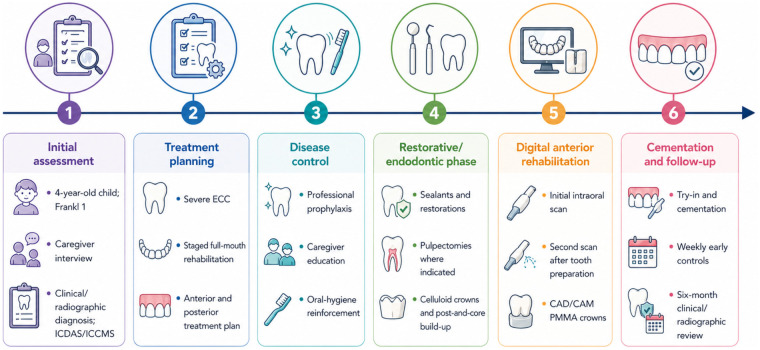
Timeline of diagnosis, treatment, digital workflow, and follow-up. The figure summarizes the staged clinical sequence from initial assessment and treatment planning to disease control, restorative/endodontic management, digital anterior rehabilitation with CAD/CAM PMMA crowns, cementation, and follow-up. CAD/CAM, computer-aided design/computer-aided manufacturing; ECC, early childhood caries; ICDAS, International Caries Detection and Assessment System; ICCMS, International Caries Classification and Management System; PMMA, polymethyl methacrylate.

Treatment first focused on disease control through professional prophylaxis, caregiver education, oral-hygiene reinforcement, and preventive/restorative care according to tooth-level needs. Severely affected teeth were managed with pulpectomy when indicated, followed by post-and-core reconstruction and crown placement. For the maxillary anterior segment, a digital pathway was incorporated to support definitive rehabilitation, including an initial intraoral scan for documentation and planning and a second scan after tooth preparation. The anterior PMMA crowns were then designed and fabricated using CAD/CAM, followed by try-in, cementation, and occlusal refinement. Clinical monitoring included early weekly controls and a six-month clinical and radiographic follow-up (Figure 2).

## Therapeutic intervention

A digitally assisted approach was selected because the patient's limited cooperation (Frankl 1) made conventional impression-taking impractical and threatened completion of definitive rehabilitation. Behavioral management relied on non-pharmacological techniques, primarily a tell–show–do approach, to improve tolerance across visits. Treatment was therefore staged to minimize procedural burden, consolidate definitive steps, and preserve strict field control during adhesive procedures.

Full-mouth rehabilitation integrated prevention, restorative care, and endodontic therapy where indicated (Table 1). Initial visits prioritized disease control and risk reduction through professional prophylaxis, caries-control measures, and structured caregiver education focused on age-appropriate oral-hygiene technique and the need for parental supervision of daily toothbrushing. Preventive sealants were placed on posterior teeth according to the tooth-level plan, and restorative procedures were completed as needed. Multiple teeth requiring full coronal coverage were rehabilitated with celluloid crowns, and a vertical dimension adjustment was incorporated as part of the comprehensive restorative strategy to re-establish function and facilitate rehabilitation in the setting of generalized breakdown.

Given the patient's young age and the goal of maintaining the primary dentition for function and development, teeth requiring endodontic management underwent pulpectomy prior to definitive coronal coverage. When post-assisted reconstruction was indicated, post-and-core build-up was performed to provide retention and structural support. In the maxillary anterior segment, biologic posts were selected for teeth 51, 52, 61, and 62 because of the extensive coronal destruction and the need to improve retention of the definitive restorations after pulpectomy. Biologic posts fabricated from natural tooth structure have been described as a conservative and esthetic option for the reconstruction of severely damaged primary anterior teeth, offering dentin-like mechanical behavior, favorable bonding potential, and good anatomical compatibility with the prepared canal space. In the present case, the donor tooth had exfoliated naturally from the patient's sibling and had been kept by the caregiver. Because storage conditions and duration were not standardized, the tooth was managed as potentially contaminated. It was thoroughly debrided to remove adherent organic debris and steam-sterilized in an autoclave at 121 °C for 15 min, followed by drying and immediate chairside preparation to obtain passively fitting post forms. Post cementation and core build-up were performed using a dual-cure resin composite system (Paracore), following the manufacturer's instructions.

**Figure 3:**
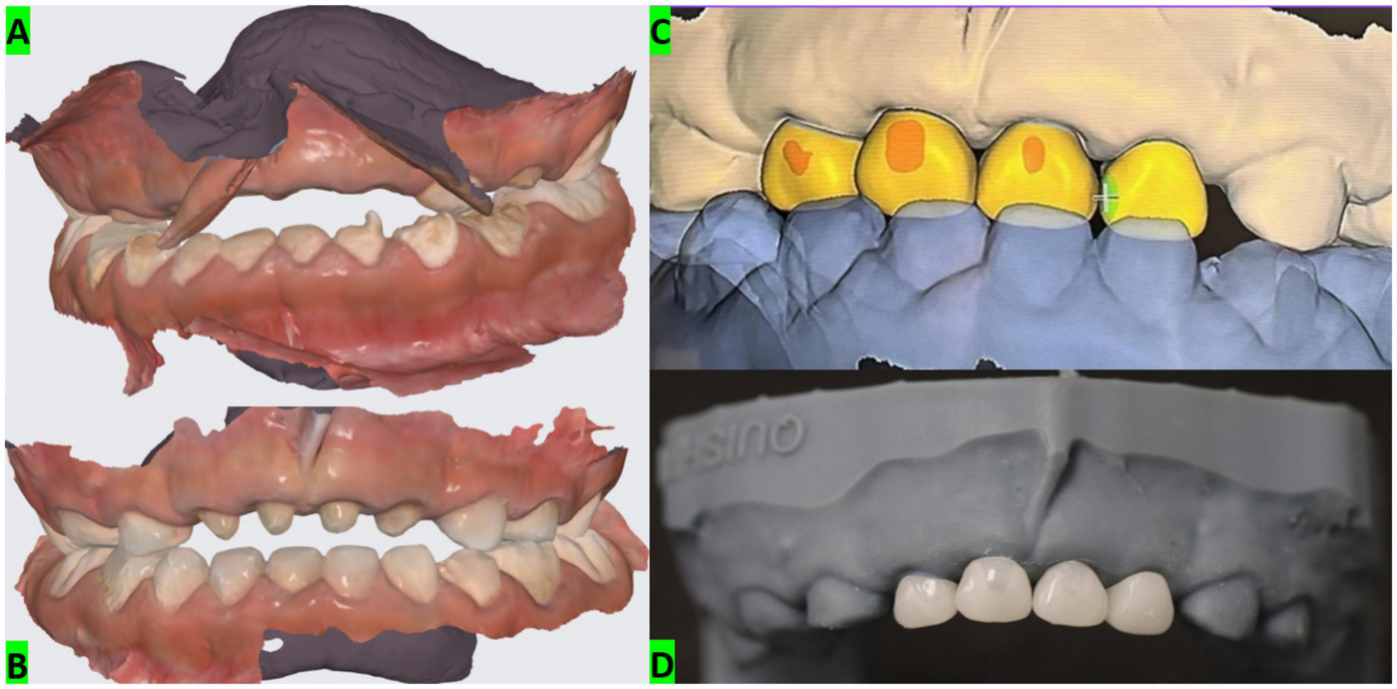
Two-step intraoral scanning and digital crown planning for anterior rehabilitation. **(A)** Initial intraoral scan acquired for baseline digital planning and assessment. **(B)** Second intraoral scan obtained after endodontic treatment and post-and-core reconstruction to capture the prepared abutments and guide definitive restoration design. **(C)** Computer-aided design view showing the planned full-coverage crowns for the primary maxillary incisors. **(D)** Fabricated anterior crowns (milled) prior to clinical try-in and cementation.

**Figure 4:**
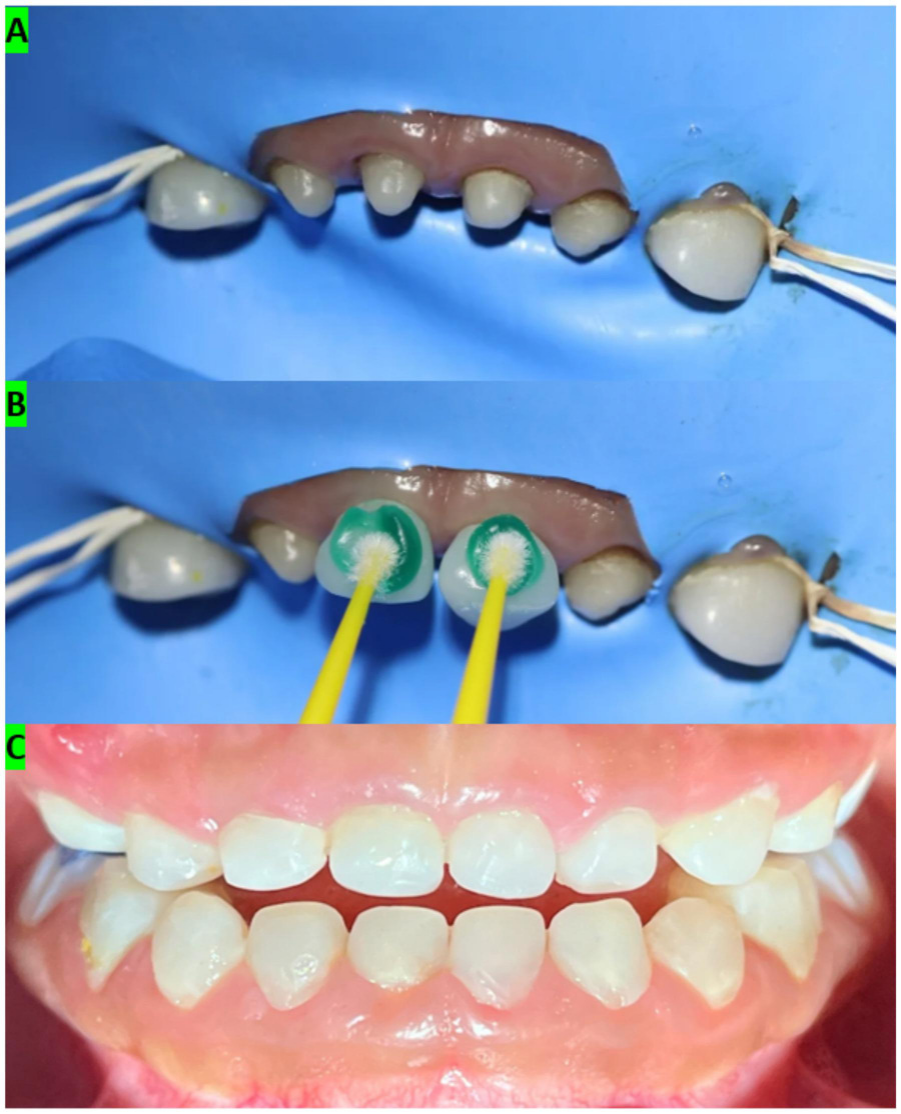
Cementation protocol and immediate clinical outcome of the anterior crowns. **(A)** Rubber dam isolation prior to cementation of the maxillary anterior crowns. **(B)** Surface conditioning/cleaning of the restorations before cementation under controlled isolation. **(C)** Immediate postoperative frontal view after cementation, showing re-established anterior form and esthetics with clinically acceptable occlusion.

For definitive anterior crown rehabilitation, a two-step digital workflow was employed. An initial intraoral scan was obtained at presentation to support diagnostic documentation and treatment planning. After completion of endodontic and restorative procedures and following tooth preparation for full coverage, a second intraoral scan was acquired to capture the reconstructed abutments and enable definitive crown design. Both arches were scanned with an intraoral scanner (Medit i700). Crowns were designed in computer-aided design (CAD) software (exocad) and manufactured by computer-aided manufacturing (CAM) milling in PMMA (Figure 3). Tooth preparation for full coverage was standardized with approximately 1.5–2.0 mm reduction and a chamfer finish line. Try-in was performed to verify marginal adaptation and occlusion, with adjustments as required. Definitive cementation was completed under rubber dam isolation using a self-adhesive resin cement (3M RelyX U200), followed by removal of excess cement and occlusal refinement (Figure 4).

Overall, this staged strategy enabled full-mouth rehabilitation within the child’s behavioral limits by avoiding conventional impressions, consolidating definitive steps, and leveraging intraoral scanning to complete CAD/CAM crown fabrication under clinically controlled conditions.

## Follow-up and outcomes

At the time of crown cementation, the patient reported no pain and no immediate complications were observed. Given the extent of rehabilitation and the initial behavioral constraints, follow-up was structured with weekly early controls and subsequent six-monthly reviews to monitor overall oral health, restoration integrity, and stability of the rehabilitated occlusion. Clinical surveillance focused on retention and integrity of both PMMA and celluloid crowns, marginal adaptation, gingival response, plaque control, and functional parameters including occlusal contacts and comfort. Radiographic monitoring with periapical assessment was incorporated to evaluate periapical status and to screen for resorptive changes over time in endodontically treated teeth.

At the first six-month follow-up, clinical and radiographic findings were satisfactory. The PMMA crowns remained intact with stable margins, gingival tissues were healthy, occlusal relationships were clinically acceptable, and periapical regions were unremarkable, with no radiographic signs suggestive of pathological resorption. Given the child's age and the expected timing of physiological exfoliation, clinically relevant resorptive changes affecting post-retained restorations were not anticipated at this early stage; nevertheless, continued surveillance was planned. A longitudinal objective is to document the behavior of post-retained anterior restorations over time, with the incisors restored with biologic posts (51, 52, 61, and 62) monitored at six-month intervals to assess function and to identify any emerging resorptive patterns as the patient approaches the mixed-dentition transition. Progressive adaptation to the dental setting was also observed as treatment advanced, facilitating maintenance visits and ongoing preventive care.

## Discussion

This case illustrates a behavior-driven indication for a digital workflow in pediatric dentistry. In a four-year-old child with severe ECC and very limited cooperation (Frankl 1), conventional impression-taking was not tolerated and became a practical barrier to definitive rehabilitation. In this context, intraoral scanning was not used merely as a technological alternative to conventional impressions. Rather, it functioned as an enabling strategy that allowed completion of staged full-mouth rehabilitation, including definitive anterior CAD/CAM PMMA crowns, under clinically controlled conditions.

The originality of this case lies in the clinical context in which the digital workflow was applied. Intraoral scanning has been increasingly reported in pediatric dentistry, particularly for improving comfort, reducing gag reflex, and increasing child preference compared with conventional impressions (5–7). However, many available studies focus on patient-reported experience, chairside time, or technical feasibility. The present case adds a more specific perspective: digital technology was used to overcome a behavioral limitation that threatened treatment completion in a child with extensive ECC. This distinction is important because, in highly uncooperative patients, the clinical value of intraoral scanning may depend less on speed and more on tolerability, feasibility, and continuity of care.

Published pediatric reports have described digital workflows for space maintainers, customized crowns, and CAD/CAM restorations in primary teeth. Vij et al. (8) used digital impressions to fabricate a space maintainer, emphasizing improved compliance in a pediatric patient. Pelagalli et al. (9) proposed the “Kids Digital Crown Technique” as an individualized digital approach for restoring primary teeth. Case-based evidence has also supported the feasibility of digital workflows for restoring endodontically treated primary teeth, including intraoral scanning, digital design, and milling for primary molar rehabilitation (10). More recent case reports similarly suggest that CAD/CAM-based workflows can be feasible in pediatric settings, with emphasis on adaptability and acceptability (11). The present case is consistent with these reports, but differs by placing the behavioral indication at the center of the workflow, rather than presenting digital dentistry only as a restorative or esthetic upgrade (8, 9).

The reconstruction of the maxillary anterior segment also required post-assisted rehabilitation because of severe coronal destruction after pulpectomy. In this case, biologic posts were selected for teeth 51, 52, 61, and 62 to provide intracanal retention and support for the coronal build-up before definitive crown placement. Biological restorations and biologic posts have been described in pediatric dentistry as conservative and esthetic options for severely destroyed primary anterior teeth. Their proposed advantages include anatomical compatibility, dentin-like mechanical behavior, favorable esthetics, and adaptation of natural tooth structure to the prepared canal space (12, 13). However, the available evidence remains limited and is based mainly on case reports, small clinical studies, and narrative reviews. Therefore, the use of biologic posts in this case should be interpreted as a clinically justified, case-specific decision rather than as evidence of superiority over other post systems.

The decision to use biologic posts was grounded in the extent of anterior coronal destruction, the need for additional retention, and the availability of a naturally exfoliated donor tooth from the patient's sibling. This approach was considered appropriate because the maxillary anterior teeth had insufficient remaining structure to support definitive esthetic rehabilitation without post-assisted reconstruction. Nevertheless, biologic posts should not be considered universally applicable. Their use depends on donor material availability, caregiver consent, sterilization procedures, adhesive protocol control, and longitudinal monitoring. For this reason, the technique should be presented as one component of a tailored rehabilitation strategy rather than as a standard substitute for fiber posts or other intracanal retention systems.

Several strengths increase the clinical relevance of this report. First, the case reflects real-world ECC management by integrating prevention, caregiver education, sealants, conventional restorative procedures, pulpectomies, post-and-core reconstruction, and digitally manufactured anterior crowns. Second, lesion severity was documented using ICDAS and ICCMS radiographic scoring, improving clinical interpretability. Third, the report includes early clinical and radiographic follow-up, allowing initial evaluation of restoration integrity, gingival response, occlusion, and periapical status. This is relevant because much of the pediatric digital dentistry literature emphasizes comfort and preference more than post-treatment clinical outcomes.

Important methodological limitations should temper interpretation. This is a single case report and cannot establish superiority of the digital workflow over conventional approaches. The favorable outcome may have been influenced by case selection, operator experience, caregiver adherence, laboratory support, and the controlled clinical setting. In addition, the six-month follow-up provides only an early assessment. Although the PMMA crowns remained intact and radiographic findings were satisfactory, this period is too short to support conclusions about medium- or long-term restoration stability. Longer follow-up is required to evaluate restoration survival, marginal integrity, occlusal changes, periapical health, and the behavior of post-retained restorations during physiological root resorption and transition toward mixed dentition.

Implementation limitations are also relevant. Intraoral scanners, CAD/CAM software, milling units, laboratory support, and trained personnel may not be available in all pediatric dental settings. Cost may further restrict adoption, especially in resource-limited or publicly funded clinical settings. These barriers are important because ECC is common in populations where access to advanced digital infrastructure may be limited. Therefore, the applicability of this workflow depends not only on clinical indication, but also on local resources, professional training, and the capacity to maintain follow-up.

For clinical practice, this case supports considering intraoral scanning as a targeted strategy when conventional impressions are not tolerated (12, 13). The digital step, however, does not replace the fundamentals of pediatric restorative care. Disease control, caregiver education, field isolation, fit verification, adhesive protocol control, cementation quality, and structured follow-up remain essential. The main clinical implication is therefore not that digital workflows should replace conventional approaches, but that they may expand treatment options in selected children with behavioral limitations and extensive restorative needs.

From a broader health-care perspective, this approach may be relevant if it reduces failed clinical steps, repeated impression attempts, or incomplete rehabilitation in behaviorally challenging children. However, any potential benefit must be weighed against equipment costs, training requirements, and unequal access to digital technology. Future research should move beyond simple comparisons of digital and conventional impressions. Studies should stratify children by behavioral profile, report treatment completion rates, repeated attempts, chairside time, patient and caregiver experience, cost-effectiveness, and 12–24-month clinical and radiographic outcomes after extensive ECC rehabilitation.

## Patient perspective

The patient's mother expressed high satisfaction with her child's recovered oral health and the overall treatment experience. She reported that the rehabilitation improved her child's function and appearance, which reduced her initial concern and increased her confidence in maintaining his oral health. She also stated that she has become more aware that, at this age, she must take primary responsibility for her child's daily toothbrushing and oral hygiene routine. The mother specifically appreciated the use of an innovative, digitally supported approach, noting that during previous attempts with traditional methods her child did not cooperate, whereas this treatment pathway felt more manageable and ultimately allowed the care to be completed.

## Data Availability

The original contributions presented in the study are included in the article/supplementary material, further inquiries can be directed to the corresponding authors.
